# The impact of social activities on mental health among older adults in China

**DOI:** 10.3389/fpubh.2024.1422246

**Published:** 2024-08-21

**Authors:** Duanyang Gao, Rui Li, Yuying Yang

**Affiliations:** ^1^School of Social Research, Renmin University of China, Beijing, China; ^2^Center for Sociological Theory and Methodology, Renmin University of China, Beijing, China; ^3^College of Public Administration and Humanities, Dalian Maritime University, Dalian, China; ^4^School of Agricultural Economics and Rural Development, Renmin University of China, Beijing, China

**Keywords:** social activities, social activities frequency, mental health, older adults, China

## Abstract

**Background:**

Understand the current situation of social activities among older adults and its impact on mental health, providing policy basis and intervention measures to improve the mental health of the older adult.

**Method:**

Collect relevant data from 8,181 older adults aged ≥ 60 years old from the 2020 China Health and Retirement Longitudinal Study (CHARLS), constructing an analysis framework for social activities of older adults in China from three aspects: participation in social activities, number of social activities, frequency of social activities, and describe them, then analyzing the impact of social activities on their mental health using OLS and 2SLS regression models. This study also discusses the impact of eight social activities on the mental health of older adult people through subgroup.

**Result:**

Among 8,181 older adults aged 60 and above, 3,808 participated in social activities (56.24%), average number of social activities is 0.71, and average frequency of social activities is 1.31 times. The average score of mental health level measured by CES-D is 15.24 points. Participate in social activities can reduce the depression level of the older adult (β = −0.352, 95%CI: −0.547~−0.158); the more social activities the older adult participate in, the lower the depression level of the older adult (β = −0.214, 95%CI: −0.313~−0.115); the higher the frequency of participating in social activities, the lower the depression level of the older adult (β = −0.133, 95%CI: −0.182~−0.083). In summary, social activities can improve the mental health level of the older adults in China. As for different types of social activities, visiting and socializing with friends, participating in club organization activities, playing mahjong, chess, cards, or going to community activity rooms and attending school or training courses can improve the mental health of the older adult.

**Discussion:**

The social activities in three dimensions among older adults in China need to be further improved, and participating in social activities can help improve their mental health. The conclusion provides important policy implications for encouraging and supporting older adults to participate in various social activities, increasing the number and frequency of older adult social activities, then help improving the mental health level of older adults.

## 1 Introduction

According to the China’s seventh national population census in 2020, the total older adult people aged 65 and above and their proportion to the national population were 190.64 million and 13.5%, respectively, the aging of the population has further deepened compared to the previous population census (1). According to statistic of National Bureau of Statistics in 2023, the total older adult people aged 65 and above and their proportion to the national population have reached 217.09 million and 15.4%, respectively, meeting the standard of a moderately aging society (2). The deepening of the degree of aging has posed new challenges to economic development, social governance and intergenerational harmony, including a decline in labor and productivity (3), an increase in social burden and social security expenditure pressure (4), and greater barriers to intergenerational communication and understanding (5). At the same time as the aging population accelerates, mental health issues among the older adult have become increasingly prominent. The “Report on National Mental Health Development in China” (released in 2021) pointed out that depression is one of the common psychological symptoms among older adult people in China. About one-fifth (19.05%) of older adult people are in a mild depression state, and nearly one tenth (12.17%) of older adult people have moderate to high levels of depression. And depression is very common, with no significant differences in depression scores among older adult people of different genders, educational levels, marital status, and family structures (6). Long term depression can harm the mental health of the older adult, leading to the onset of depression and even further developing into serious situations such as self-harm and self-injury (7, 8).

Therefore, actively responding to population aging and its impact has risen to a national strategy. Since 2021, the Chinese government has released a series of documents to further implement its national strategy to address population aging, aiming at promoting social participation among the older adult. For example, the “Opinions on Strengthening Aging Work in the New Era” points out that high-quality social participation of the older adult should be achieved by expanding the supply of older adult education resources, improving the quality of older adult cultural and sports services, and encouraging the older adult to continue to play their role (9). “The 14th Five-Year Plan for the Development of National Aging Industry and Elderly Care Service System” highlights that it is necessary to guide the older adult to actively participate in family, community, and social development according to their own situation, and support their participation in civilized practice, public welfare and charity, volunteer service, science, education, culture, and health undertakings (10). It can be seen that the relevant documents emphasize the promotion of social participation among the older adult, enhancing their sense of achievement, happiness, and security, and improving their mental health.

The social activities of older adults and its impact on mental health has been extensively discussed in previous studies. A sample of 213,332 older adults (55+) was collected in the region of South Limburg in the Netherlands and the statistics found that 56% of older people are involved in social activities (11). Another nationally representative longitudinal survey of older Koreans showed that 62.6% participated in social clubs or leisure, cultural, or sports groups, 25.8% participated in alumni associations, hometown associations, or family councils, and 4.6% participated in volunteer groups, political parties, non-governmental organizations, or interest groups (12). Based on data from the Swiss Household Panel, the number of social activities that older adult participate in is 2.5 in 2013 and 2.7 in 2016, the most popular activities in both years are meeting with friends and meeting children, followed by being in a club or group (13). Developing countries, such as Brazil, have lower rates of participation in social activities among older persons (25.1%) (14). At the same time, a number of studies have worked to reveal the correlation between socialization and mental health in older adults. For example, a survey of 32,715 adults aged 50 years or older from six developing countries, including China, Ghana, India, and Mexico, found that social participation reduces the risk of mild cognitive impairment (MCI) and is beneficial to mental health (15). Participation in external social activities, including charity/volunteer work, sports/social clubs, educational/training course, and political/community organizations, contributes to the reduction of loneliness among older Europeans (16). Social participation can play a role in buffering against the negative implications of physical health decline for mental health among older adult (17).

For the effect of social activities on mental health among older adults, relevant studies have put forward many enlightening research conclusions. However, most previous studies have tended to portray social activities from a single perspective, such as whether or not participate in social activities and the number of social activities, and lack a comprehensive examination. There is also a lack of examination of the impact of more types of social activities. The above deficiencies may affect the presentation of social activities for older adults and further affect the precision of policy development.

Therefore, the improvements of this study include the following three aspects. Firstly, this study reasonably and comprehensively examines the social participation of the older adult to analyze its impact on mental health. Not only examines whether older adult people participate in social activities, the frequency of social activity participation, and the quality of social activity participation, but also conducts regression analysis from eight different types of social activities. The comprehensive examination and reasonable division have further strengthened the scientificity and innovation. Secondly, focus to the impact of social activities on the mental health of the older adult population in China, provide new ideas for improving the mental health level of the older adult, and provide decision-making basis for China and other countries facing serious population aging problems to actively address this issue. Finally, this study effectively addressed endogeneity issues using the 2SLS regression model, which more accurately identified the relationship between social activities and the mental health of the older adult population.

In conclusion, former studies have found that social activities are associated with mental health of the older adults, but further research is needed to improve. Therefore, based on the above analysis, the purposes of this study are: (1) to analyze the current situation of social activities among older adult people in China; (2) to find whether social activities have an impact on the mental health of the older adult through the system of social activity participation, social activity quantity, and social activity frequency; and (3) to explore which social activities have a greater impact on the mental health of the older adult.

## 2 Materials and methods

### 2.1 Data

Data for this study were collected from the 2020 China Health and Retirement Longitudinal Study (CHARLS). The survey collected data on the middle-aged and older adult population aged 45 and above from 450 village committees in 150 counties and districts across 28 provinces (autonomous regions, municipalities) in China, including community, family, and individual questionnaires, covering basic information, income, work, health status, and older adult care, and has good representativeness for the older adults in China (18). This survey is the fifth round of the CHARLS national survey, with approximately 11,377 households and 19,395 individuals collected as valid samples at the household level. Our study selected older adults aged ≥ 60 years old, and after processing for missing values in the sample, 8,181 samples were collected. The collected content includes social activity status (participation, number of social activities, frequency of social activities), mental health status, and control variables (gender, age, education level, marital status, place of residence, economic status, availability of medical insurance, adoption of older adult care insurance, self-assessment of health status, and daily activity ability ADL).

### 2.2 Assessment of mental health

Depression is a commonly used core indicator reflecting mental health status and is widely used in research on the mental health of the older adult (19, 20). CHARLS 2020 uses the Center for Epidemiological Studies Depression Scale (CES-D) to assess the mental health level of older adults (21). The scale includes 10 items, of which 8 are negative emotional issues, including whether to worry about small things, difficulty concentrating, low mood, difficulty doing things, feeling scared, poor sleep, loneliness, and inability to continue living. Two are positive emotional issues, including being hopeful for the future and feeling happy. All 10 items are scored at 4 points, with very little or no (<1 day) assigned a score of 0 points, not much (1–2 days) assigned a score of 1 point, sometimes or half of the time (3–4 days) assigned a score of 2 points, and most of the time (5–7 days) assigned a score of 3 points. It should be noted that the two positive emotional issues are scored in reverse, and then the scores of the 10 items are summed up to construct a comprehensive depression index for the older adult, with a value range of 0–30 points. The internal consistency coefficient (Cronbach’s α) is 0.724, which is greater than 0.7, indicating high data reliability and reliability of measurement results. The higher the CES-D score, the more severe the depression level in the older adult, indicating a lower level of mental health in the older adult. The average score of 8,181 older adults aged 60 and above is 15.24 points.

### 2.3 Independent variables and other variables

Social activities, including social activities participation, social activities number and social activities frequency, are set according to the questions provided in the CHARLS questionnaire.

Firstly, social activities participation. According to “Have you engaged in the following social activities in the past month? (Multiple choices).” A total of 9 options have been set, including (1) Visiting or socializing with friends; (2) Playing mahjong, chess, cards, or going to community activity rooms; (3) Providing assistance to relatives, friends, or neighbors who do not live with; (4) Dancing, fitness, practicing qigong, etc.; (5) Participating in club organization activities; (6) Volunteer activities, charitable activities, taking care of patients or people with disabilities who do not live with; (7) Attending school or training courses; (8) Other social activities; (9) None of the above. If a respondent chooses any one of the first eight options, it means that the respondent has participated in social activities (assigned a value of 1). If a respondent chooses the ninth option, it means that the respondent has not participated in social activities (assigned a value of 0).

Secondly, social activities number. Due to the above question is a multiple-choice question, the respondents’ selected social activities can be aggregated to obtain the number of social activities for the older adult.

Finally, social activities frequency. If the older adult chooses the first eight social activities, they need to further answer “How often did you engage in the activities mentioned earlier in the past month?” The options include “infrequent,” “almost weekly,” and “almost daily.” According to the practice of existing research (22), the option “infrequent” is assigned a score of 1, “almost weekly” is assigned a score of 2, and “almost daily” is assigned a score of 3. Multiplying each selected social activity by its score and then adding them up can yield the frequency of social activities among older adult people in China.

In addition, in order to improve the accuracy of regression estimation, this study also sets control variables based on previous research and questionnaire questions. Studies have found that women have lower levels of mental health (23). With age, physical function declines and older people are prone to depression; education level shows a linear correlation with mental health, and experiences such as widowhood are more likely to worsen mental health in older people (24, 25). However, it has also been noted that the mental health of older adults is better than that of younger adults (26). Overall, gender, age, education level and marital status are correlated with the mental health of older persons. In China, older adults living in rural areas are more likely to have lower levels of mental health due to the objective existence of urban–rural differences (27). There is a consensus that economic income affects mental health, and in general, the higher the income level, the higher the level of mental health of older persons (28). Having health insurance and receiving a pension can provide protection for the older adult after retirement, serving the function of “eliminating worries” and preventing them from falling into depression (29, 30). A regression discontinuity design study found that Chinese older adults who received pensions were able to improve their mental health and further improve their health behaviors (31). In addition, physical health is a factor that should not be overlooked in the mental health of older adults, and physical health is positively associated with mental health in older adults (32, 33).

Therefore, the control variables selected for this study and their definitions have been identified, including gender (male = 1, female = 0), age, marital status (in marriage = 1, not in marriage = 0), education level (middle school and above = 1, primary school and below = 0), place of residence (city = 1, rural = 0), economic status (quartile), endowment insurance (participation = 1, not participation = 0), medical insurance (participation = 1, not participation = 0), self-assessment of health status (“What do you think of your health status?,” very good = 5, good = 4, general = 3, bad = 2, very bad = 1). ADL in daily activity ability (whether there are difficulties in 12 daily activities such as dressing, bathing, eating, getting up and out of bed, going to the toilet, controlling urination and defecation, doing household chores, cooking, shopping, making phone calls, taking medication, and managing money). Daily activity ability (ADL) is scored using a 4-point scale for each item (no difficulty = 1, difficult but still able to complete = 2, difficult, need help = 3, unable to complete = 4). All items in the scale that are not difficult and can be completed independently are considered normal for ADL, and at least one item is defined as difficult but still able to complete, difficult, in need of help or unable to complete.

### 2.4 Statistical analysis

This study uses Stata 16.0 statistical software for general descriptive analysis χ 2-test, using ordinary least squares estimation (OLS) to observe the relationship between social activity and mental health of older adults in China. It should be noted that factors such as omitted variables and reverse causal relationships may lead to potential endogeneity of social activities in the mental health. Therefore, instrumental variables are needed to address endogeneity issues. This study used two-stage least squares (2SLS) to control for endogeneity based on ordinary least squares (OLS). In the first stage, the method used endogenous explanatory variables to regress the instrumental variables, separating the exogenous part of the endogenous explanatory variables. In the second stage, the dependent variable was used to regress the fitted values of the first stage regression. The advantages of two-stage least squares (2SLS) include the ability to handle endogeneity issues, no restrictions on the distribution of variables, and the ability to use both normal and non-normal distributions. The instrumental variables selected in this study will be discussed in detail in the “Discussion on Endogeneity.”

## 3 Results

This section may be divided by subheadings. It should provide a concise and precise description of the experimental results, their interpretation, as well as the experimental conclusions that can be drawn.

### 3.1 General situation

Among 8,181 older adults aged 60 and above in China, there are 4,105 males (50.18%) and 4,076 females (49.82%). The average age is 67.98 years old, of which 5,387 people (65.85%) are aged 60 to 69 years old, accounting for more than 60% of young older adults. 5,851 people (71.52%) have a primary school education or below, and 2,330 people (28.48%) have a junior high school education or above. 1,673 people (20.45%) are not currently married, and 6,508 people (79.55%) are currently married. 2,874 people (35.13%) currently reside in urban areas and 5,307 people (64.87%) in rural areas. The average annual income is 12434.23 yuan. 1,059 people (12.94%) did not receive pension insurance benefits, and 7,122 people (87.06%) received pension insurance benefits. 384 people (4.69%) have no medical insurance, 7,797 people (95.31%) have medical insurance. Among the self-rated health status, 584 people (7.14%) are unhealthy, 1,678 people (20.51%) are average, 4,130 people (50.48%) are relatively healthy, 933 people (11.40%) are very healthy, and 856 people (10.46%) are very healthy. The average score for self-care level is 13.79 points, and overall, older adults have a higher level of self-care.

### 3.2 Current situation of social activities among older adults in China

As shown in Table 1, among 8,181 older adults aged 60 and above, 3,808 participated in social activities, accounting for 46.55%, while 4,373 did not participate in social activities, accounting for 53.45%. The average number of social activities among older adults is 0.71, with a frequency range of 0–14 and an average frequency of 1.31 social activities. Among the 3,808 older adults who participated in social activities, 2,448 (64.29%) visited and socialized with friends, 1,306 (34.30%) played mahjong, chess, cards, and went to community activity rooms, 952 (25.00%) assisted to relatives, friends, or neighbors who did not live with you, 480 (12.61%) participated in dance, fitness, qigong, and other activities, 213 (5.59%) participated in club activities, 178 (4.67%) participated in volunteer activities, charity activities, or caring for patients or disabled people who did not live with you, 58 (1.52%) attended school or training courses, and 154 (4.04%) participated in other social activities. Taking social activity participation as an example to compare different characteristics of Chinese older adults, it was found that the social activity participation of older adults with different ages, education levels, current residence, income situation, pension insurance, self-rated health status, and ADL level of self-care were different, and the differences were statistically significant (all *p* < 0.001).

**Table 1:** Social activities of older adults with different characteristics in China.

Variables		Total	Social activities participation		χ^2^	*P*-value
			Total	Percentage/%		
Gender	Male	4,105	1,885	45.92	1.303	0.254
	Female	4,076	1,923	47.18		
Age	60~69	5,387	2,585	47.99	13.12	<0.001
	≥70	2,794	1,223	43.77		
Education	Primary school and below	5,851	2,547	43.53	75.100	<0.001
	Middle school and above	2,330	1,261	54.12		
Marriage	Not in marriage	2,330	792	33.99	0.532	0.466
	In marriage	5,851	3,016	51.55		
Residence	Rural	5,307	2,307	43.47	57.449	<0.001
	City	2,874	1,501	52.23		
Income (quartile)	0%~25%	1,745	694	39.77	103.664	<0.001
	25%~50%	2,259	990	43.82		
	50%~75%	2,117	983	46.43		
	75%~100%	2,060	1,141	55.39		
Endowment Insurance	Participation	1,059	443	41.83	10.869	<0.001
	Not participation	7,122	3,365	47.25		
Medical Insurance	Participation	384	151	39.32	8.451	0.004
	Not participation	7,797	3,657	46.90		
Health Status	Very bad	584	217	37.16	31.005	<0.001
	Bad	1,678	746	44.46		
	General	4,130	1,993	48.26		
	Good	933	458	49.09		
	Very good	856	394	46.03		
ADL	Normal	5,024	2,413	48.03	11.502	<0.001
	Limited	3,157	1,395	44.19		

### 3.3 Impact of social activities on mental health of older adults

When controlling for a set of control variables, Table 2 shows the OLS results that social activities participation, social activities number, and social activities frequency are significantly correlate with the mental health of older adults in China. Compared with older adult people who did not participate in social activities, those who participated in social activities scored lower in depression, with a coefficient of-0.352, 95% CI of (−0.547, −0.158), and *p* = 0.000. The more social activities the older adult participate in, the lower their depression scores, with a coefficient of-0.214, 95% CI of (−0.313,−0.115), and *p* = 0.000. Furthermore, the higher the frequency of social activities, the lower the depression score of the older adult, with a coefficient of-0.133, 95% CI of (−0.182, −0.083), and *p* = 0.000. Besides, gender, age, education, marriage, residence, medical insurance, health status, and ADL all have statistical significance (*p* ≤ 0.01).

**Table 2:** OLS results of the impact of social activities on mental health of older adults in China.

Variables	Coefficient (S.E.)	95% CI	*P*-value	Coefficient (S.E.)	95% CI	*P*-value	Coefficient (S.E.)	95% CI	*P*-value
Social activities participation	−0.352^***^ (0.099)	(−0.547, −0.158)	0.000						
Social activities number				−0.214^***^ (−0.05)	(−0.313,-0.115)	0.000			
Social activities frequency							−0.133^***^ (−0.025)	(−0.182, −0.083)	0.000
Gender	−0.849^***^ (−0.105)	(−1.055,−0.644)	0.000	−0.851^***^ (−0.105)	(−1.056,-0.645)	0.000	−0.863^***^ (−0.105)	(−1.069, −0.657)	0.000
Age	−0.028^***^ (−0.009)	(−0.045,-0.011)	0.001	−0.029^***^ (−0.009)	(−0.046,-0.012)	0.001	−0.028^***^ (−0.009)	(−0.045, −0.011)	0.001
Education	−0.853^***^ (−0.117)	(−1.083, −0.623)	0.000	−0.826^***^ (−0.118)	(−1.057,-0.594)	0.000	−0.831^***^ (−0.118)	(−1.061, −0.600)	0.000
Marriage	−1.229^***^ (−0.137)	(−1.499, −0.960)	0.000	−1.226^***^ (−0.137)	(−1.495,-0.957)	0.000	−1.239^***^ (−0.137)	(−1.508, −0.970)	0.000
Residence	−0.683^***^ (−0.113)	(−0.904, −0.463)	0.000	−0.674^***^ (−0.113)	(−0.895, −0.453)	0.000	−0.659^***^ (−0.113)	(−0.880, −0.438)	0.000
Income (quartile)	0.092 (−0.153)	(−0.208, 0.391)	0.549	0.099 (−0.153)	(−0.201, 0.399)	0.516	0.098 (−0.153)	(−0.201, 0.398)	0.520
Endowment insurance	−0.325 −0.243()	(−0.802, 0.152)	0.182	−0.33 (−0.244)	(−0.807, 0.148)	0.176	−0.337 (−0.243)	(−0.814, 0.141)	0.167
Medical insurance	−0.523^***^ −0.052()	(−0.625, −0.421)	0.000	−0.519^***^ (−0.052)	(−0.621, −0.417)	0.000	−0.516^***^ (−0.052)	(−0.617, −0.414)	0.000
Health status	−1.405^***^ (−0.053)	(−1.508, −1.301)	0.000	−1.402^***^ (−0.053)	(−1.506, −1.299)	0.000	−1.402^***^ (−0.053)	(−1.505, −1.298)	0.000
ADL	2.363^***^ (−0.119)	(2.130, 2.596)	0.000	2.360^***^ (−0.119)	(2.127, 2.592)	0.000	2.353^***^ (−0.119)	(2.121, 2.586)	0.000
Constant	24.054^***^ (−0.710)	(22.661,25.446)	0.000	24.061^***^ (−0.710)	(22.669, 25.453)	0.000	24.015^***^ (−0.709)	(22.625, 25.404)	0.000
*R^2^*	0.272			0.273			0.273		

S.E., Standard Error; CI, confidence interval; ^*^p < 0.1, ^**^p ≤ 0.05, ^***^p ≤ 0.01.

### 3.4 Discussion on endogeneity

The above results have shown that social activities can help improve the mental health of older adults in China. However, this conclusion may have a reverse causal relationship, meaning that psychologically healthy Chinese older adult are more likely to engage in social activities. The instrumental variable method addresses endogeneity issues to overcome this potential reverse causal interference. The average probability of older adults in the community (village) participating in social activities other than the respondents themselves is used as the instrumental variable. From the perspective of correlation, the social activities of older adult individuals are influenced by the social activities of other older adults in the surrounding community (village); from the perspective of exogeneity, whether other older adults in the community (village) where the older adult reside engage in social activities does not directly affect their mental health, that is, meeting the requirements of instrumental variable correlation and exogeneity selection. According to Table 3, in the first stage regression of two-stage least squares estimation, the coefficient of the instrumental variable is significantly positive at the 1% statistical level, indicating that the selection of the instrumental variable meets the correlation requirements. In the second stage regression of two-stage least squares estimation, the coefficient of the impact of social activity participation on the mental health of older adults in China is a negative 0.928, which is consistent with the regression conclusion estimated by the least squares method. Similarly, the two-stage minimum quadratic estimation results of the number of social activity items and social activity frequency are also consistent with the conclusion drawn from Table 2. Social activities have indeed improved the mental health of older adults in China. In terms of various statistical test indicators, the *p*-value is 0.000, rejecting the hypothesis of exogeneity at the 1% level, indicating that social activity participation, number of social activity items, and social activity frequency are endogenous variables. The F-statistics are all greater than 10, which can exclude the problem of weak instrumental variables.

**Table 3:** 2SLS results of the impact of social activities on mental health of older adults in China.

Estimated	Instrumental variable		Social activity participation		Control variables	Constant		*P*-value	F
	Coefficient	S.E.	Coefficient	S.E.		Coefficient	S.E.		
Phase 1	0.894^***^	(0.000)			Yes	0.204	(0.075)	0.000	888.189
Phase 2			−0.928^**^	(−0.006)	Yes	24.308	(0.728)		
**Estimated**	**Instrumental variable**		**Social activities number**		**Control variables**	**Constant**		***p*-value**	**F**
	**Coefficient**	**SE**	**Coefficient**	**SE**		**Coefficient**	**SE**		
Phase 1	1.529^***^	(0.059)			Yes	0.354	(0.133)	0.000	668.686
Phase 2			−0.543^**^	(0.198)	Yes	24.310	(0.729)		
**Estimated**	**Instrumental variable**		**Social activities frequency**		**Control variables**	**Constant**		***p*-value**	**F**
	**Coefficient**	**SE**	**Coefficient**	**SE**		**Coefficient**	**SE**		
Phase 1	2.829^***^	(0.118)			Yes	0.122	(0.282)	0.000	568.508
Phase 2			−0.293^**^	(0.107)	Yes	24.153	(0.718)		

S.E., Standard error; ^*^p < 0.1, ^**^p ≤ 0.05, ^***^p ≤ 0.01.

### 3.5 Heterogeneity analysis

This study then conducted subgroup analyses of the impact of the eight different types of social activities on mental health, as shown in Table 4. Type 1 to Type 8 represent the eight social activities introduced in the Independent Variables and Other Variables sections. The results showed that Type 1 (visiting and socializing with friends), Type 2 (playing mahjong, chess, cards, or going to community activity rooms), Type 5 (participating in club organization activities), Type 7 (attending school or training courses), and Type 8 (other social activities), these five types of social activities can reduce depression in the older adult and improve their mental health, with coefficient of-0.277, −0.217, −0.233, −0.147 and-0.084. Furthermore, in terms of comprehensive coefficients and significance, Type 1 (visiting and socializing with friends) can greatly improve the mental health level of the older adult, followed by Type 5 (participating in club organization activities) and Type 2 (playing mahjong, chess, cards, or going to community activity rooms). Providing community wide activities is of great significance and policy implications for improving the mental health of the older adult.

**Table 4:** Heterogeneity analysis of the impact of social activities on mental health of older adults in China.

Variables	(1)	(2)	(3)	(4)	(5)	(6)	(7)	(8)
Type 1	−0.277^***^ (0.106)							
Type 2		−0.217^***^ (0.062)						
Type 3			0.003 (0.052)					
Type 4				−0.077 (0.052)				
Type 5					−0.233^***^ (0.053)			
Type 6						−0.018 (0.058)		
Type 7							−0.147^**^ (0.067)	
Type 8								−0.084^**^ (0.041)
Constant	23.967^***^ (0.710)	23.986^***^ (0.710)	23.893^***^ (0.710)	23.921^***^ (0.709)	23.863^***^ (0.708)	23.897^***^ (0.709)	23.910^***^ (0.709)	23.881^***^ (0.709)
Control variables	Yes	Yes	Yes	Yes	Yes	Yes	Yes	Yes
*R^2^*	0.272	0.272	0.271	0.271	0.272	0.271	0.271	0.271
*N*	8,181	8,181	8,181	8,181	8,181	8,181	8,181	8,181

^*^p < 0.1, ^**^p ≤ 0.05, ^***^p ≤ 0.01; Independent variables and other variables has already pointed out that the ninth type (None of the above) is not participating in social activities, so it is not discussed here; Due to space constraints, 95% CI and *p*-value could not be reported and can be obtained from the authors.

## 4 Discussion

Social activities has been found to help improve mental health in older adults and could be an indicator of successful aging (34, 35). Former studies concentrate more on the participation in social activities and their impact on mental health, neglecting to present more detailed and broader dimensions of social activities, which may not effectively promote the social participation of the older adult. This study collected the relevant data of 8,181 older adult people aged 60 years and older from the China Health and Retirement Longitudinal Study (CHARLS) in 2020, and analyzed the current status of social activities among older adults in China and their impact on older adults’ mental health from the perspectives of social activities participation, social activities number, and social activities frequency, and further analyzed the impact of different types of social activities on older adults’ mental health.

First, the results of this study show that among the surveyed older adults in China, 3,808 participated in social activities, accounting for 46.55%. The average number and frequency of social activities among the older adult are only 0.71 and 1.31, respectively. Therefore, social activities among the older adult are still relatively lacking. In terms of mental health level, the average CES-D score of the older adult is 15.24 points, which is higher than the psychological health score set by relevant studies (36, 37), indicating the urgency and necessity of improving the mental health of the older adult in China.

Second, this study constructs a social activities analysis framework for Chinese older adults from three aspects: social activities participation, social activities number, and social activities frequency. It also describes the social activity situation. The results showed that the participation rate of social activities among young and older adults aged 60–69 was higher than that of middle-aged and older adults aged ≥ 70, with 47.99 and 43.77% participation rates, respectively. Mainly because as age increases, the participation of older adults in social activities is more affected by their health status, resulting in a lower proportion of social activity participation among middle-aged and older adults (38, 39). Social activity participation is more common among older adults with a junior high school education or above compared to those with a primary school education or below (54.12% vs. 43.53%), which is consistent with the existing study (40), that educated individuals are more inclined to participate in social activities. The proportion of older adults living in urban areas participating in social activities is higher than that in rural areas (52.23% vs. 43.47%), which is contrary to the results from USA and Indonesia, which showed that people living in rural communities or poorer villages are more likely to participate in social activities (41, 42). In addition to the scope and time of the survey, this may also be related to the large differences in China’s urban–rural dual structure and the more diverse forms of social activities provided by Chinese cities (43, 44). The higher the income, the higher the level of social activity participation among older adults who receive pension insurance. The reason is that high-income older adults generally have a better quality of life than low-income people, hold a more positive attitude toward life, and “older adults care” and “older adults dependence” promote “older adults action” (45, 46). 48.03% of older adults with normal levels of self-care participate in social activities, while 44.19% of older adults with limited levels of self-care participate in social activities, reflecting the negative effects of limited levels of self-care on social activities in older adults (47, 48).

Third, after controlling control variables, the analysis results based on OLS and 2SLS regression show that social activities of older adults in China have a significant improvement effect on their mental health. Chinese older adults who participate in social activities have a depression score 0.35 points lower than those who do not participate in social activities; for every additional social activity, the depression score of older adults in China decreases by 0.21 points; for every unit increase in social activity frequency among older adult Chinese, their depression score will decrease by 0.13 points. The positive effects of the above social activities on mental health are also consistent with research results (49, 50). This may be because, on the one hand, social activities strengthen the contact between older adult people and people outside of their families (51). Social support theory points out social support is individuals’ perception or experience in terms of being involved in a social group where people mutually support each other (52), this theory has also been used in behavior change research with older adults (53). Most older adults are retired or no longer participate in social labor, and their social circle is limited to a narrow space such as the family. Participating in various kinds of social activities can expand the activity space of the older adult and prompt them to perceive social support, which has a positive effect on promoting mental health (54). On the other hand, the psychological health of the older adult is directly related to biomedical factors. Lack of social activities can lead to abnormal amino acid, energy, and lipid metabolism in the body, negatively impacting on mental health (55, 56). Therefore, social activities not only enhance communication and interaction with the outside world for the older adult, allowing them to gain more social support, but also have biological effects, enabling them to maintain good mental health.

Fourth, this study conducts subgroup analyses of the impact of the eight different types of social activities on mental health and finds that not all types of social activities improve the mental health of older adults. Among them, visiting and socializing with friends, participating in club organization activities, playing mahjong, chess, cards, or going to community activity rooms and attending school or training courses can greatly improve the mental health level of the older adult. Participating in activities such as door-to-door visits and socializing with friends can enable older adults to share their emotions, enhancing their sense of social presence. Club organization and community activity rooms emphasize the importance of social activities relying on organizational and community spaces. Learning new knowledge slows cognitive decline (57).

In order to improve the mental health of the older adult, policymakers should give more encouragement and support to the social participation of the older adult. Based on the conclusions, we propose the following policy recommendations. Firstly, it is vital to raise the awareness of older adults to participate in social activities. China should highlight the positive role of social activities and their positive significance to mental health, encourage the older adult to actively participate in social activities. Secondly, based on the differences in the impact of different types of social activities on the mental health of older persons, this study recommends the construction of social activity circles for older persons based on associative organizations as well as the community, which includes diversifying the roles of older persons in existing groups and expanding the services by using existing community resources. Continuing education should also be given for the older adult adults to seek self-learning and advancement. Japan and Singapore’s efforts to promote social activities for the older adult are worth learning, including Japan’s use of community gardening to enhance connections between the older adult and their neighbors (58), and Singapore’s establishment of the National Silver Academy (NSA) in 2016 to promote lifelong learning among seniors (59). Finally, considering the differences in gender, education level, place of residence, income, social security, and other factors that older adults have in social activities, relevant departments should encourage older adults with low education levels and in rural areas to engage in social activities in a targeted manner. Efforts should be made to improve the social security system for older adult care.

However, this study also has limitations. First, this study was a cross-sectional design, thus cannot reflect the time-trend effect of social activities on the mental health of older adults. Therefore, it is necessary for a longitudinal design which may support our results. Second, the classification of social activity types mainly relies on CHARLS questionnaire question settings, and scientific classification or scale measurement is needed in the future. Third, the data in this study were subjective, which may have led to partial recall bias. This limitation also provide direction for further improvement, namely objective measurements, in subsequent research.

## 5 Conclusion

The results of this study indicate that social activities can improve the mental health of older adults in China. However, the overall social activities of older adults in China are still limited, and there is still room for improvement in terms of social activity participation, number of social activities, and frequency. Therefore, it is necessary to encourage older adults to actively participate in social activities and invest in their later years. At the same time, diversified social activities should be provided for the older adult to increase their participation in social activities comprehensively, so as to bring into play the influence of social activities on the mental health of the older adult and to promote healthy aging, which will lead to a new stage of development.

## Data Availability

Publicly available datasets were analyzed in this study. This data can be found at: http://charls.pku.edu.cn/.
